# Towards tick virome and emerging tick‐borne viruses: Protocols, challenges and perspectives

**DOI:** 10.1002/imo2.70022

**Published:** 2025-05-12

**Authors:** Run‐Ze Ye, Nuo Cheng, Yu‐Yu Li, Ning Wang, Xiao‐Yang Wang, Biao Deng, Yuguo Chen, Li‐Li Ren, Wu‐Chun Cao

**Affiliations:** ^1^ Qilu Hospital of Shandong University Jinan Shandong China; ^2^ State Key Laboratory of Pathogen and Biosecurity Academy of Military Medical Sciences Beijing China; ^3^ Institute of Pathogen Biology Chinese Academy of Medical Sciences & Peking Union Medical College Beijing China; ^4^ Research Unit of Discovery and Tracing of Natural Focus Diseases Chinese Academy of Medical Sciences Beijing China; ^5^ Institute of EcoHealth, School of Public Health, Cheeloo College of Medicine Shandong University Jinan Shandong China

## Abstract

Emerging tick‐borne viruses are considered a significant public health threat. The understanding of the tick virome has been revolutionized by high‐throughput meta‐transcriptomic sequencing, and numerous novel pathogens have been unveiled. However, effective data integration and comparative analyses have been hindered by inconsistent research protocols. In response, standardized protocols are proposed, covering field surveys, sample processing, sequencing library construction, virus assembly, phylogenetic analysis, and classification, offering a unified framework for tick‐virome research. Key challenges such as sampling representations, sequencing contamination, and classification discrepancies are also discussed. Furthermore, future perspectives on leveraging bioinformatics and machine learning to trace virus evolution, elucidate virus–tick–host interactions, and enhance surveillance in high‐risk areas are outlined in our work. Ultimately, special emphasis is placed on the vital significance of worldwide cooperation and data sharing, which is essential for refining methodologies and guiding targeted interventions to mitigate emerging tick‐borne diseases.

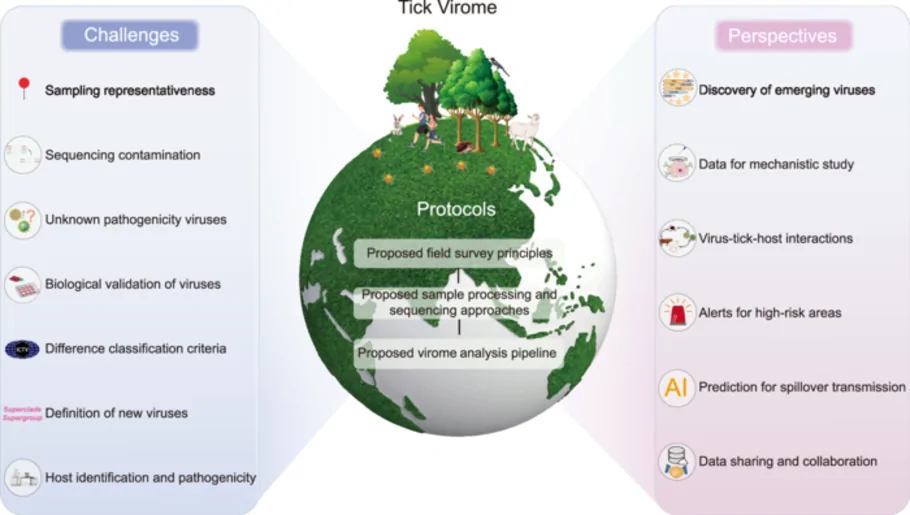

In recent years, emerging tick‐borne viruses have been increasingly identified, many of which have been reported to infect humans, posing significant threats to public health worldwide [1]. The U.S. National Institutes of Health was committed to addressing the growing public health threat posed by tick‐borne diseases (TBDs) and released the Strategic Plan for Tick‐borne Disease Research in 2019, aiming to accelerate efforts across various fields, enhance the understanding of TBDs, and promote the development of effective diagnostic, prevention, and treatment tools. Currently, multiple approaches are available to investigate tick‐borne pathogens, including traditional pathogen detection methods and high‐throughput sequencing. Research on tick‐borne viruses has also been steadily increasing (Text S1, Figure S1). Traditional pathogen detection methods can only detect known pathogens, have limited throughput, and are highly dependent on the researchers' experience with local regions and tick species, which makes the identification of emerging pathogens challenging [2]. In contrast, the high‐throughput sequencing‐based meta‐transcriptomics has greatly expanded our understanding of tick virome and promoted the identification of emerging tick‐borne viruses [3, 4]. The development of omics research has provided a critical foundation for understanding ticks and tick‐borne pathogens [5], enabling us to describe pathogen characteristics in a timely and comprehensive manner, thereby allowing targeted control of tick‐borne disease threats. However, the lack of unified research protocols and a systematic analytical framework poses challenges to the comparability of results and the integration of data across studies.

Here, we recommend a set of standardized research protocols for tick virome, including field surveys, sample processing, meta‐transcriptomic sequencing, virus assembly, phylogenetic analysis, and virus classification, aimed at gaining a comprehensive and accurate understanding of the tick virome and efficiently identifying novel viruses. These protocols will provide a scientific basis for targeted prevention and control of tick‐borne disease outbreaks. Moreover, the challenges faced in tick virome research and future research directions are discussed.

## 1 PROTOCOLS

### Proposed field survey principles

**FIGURE 1:**
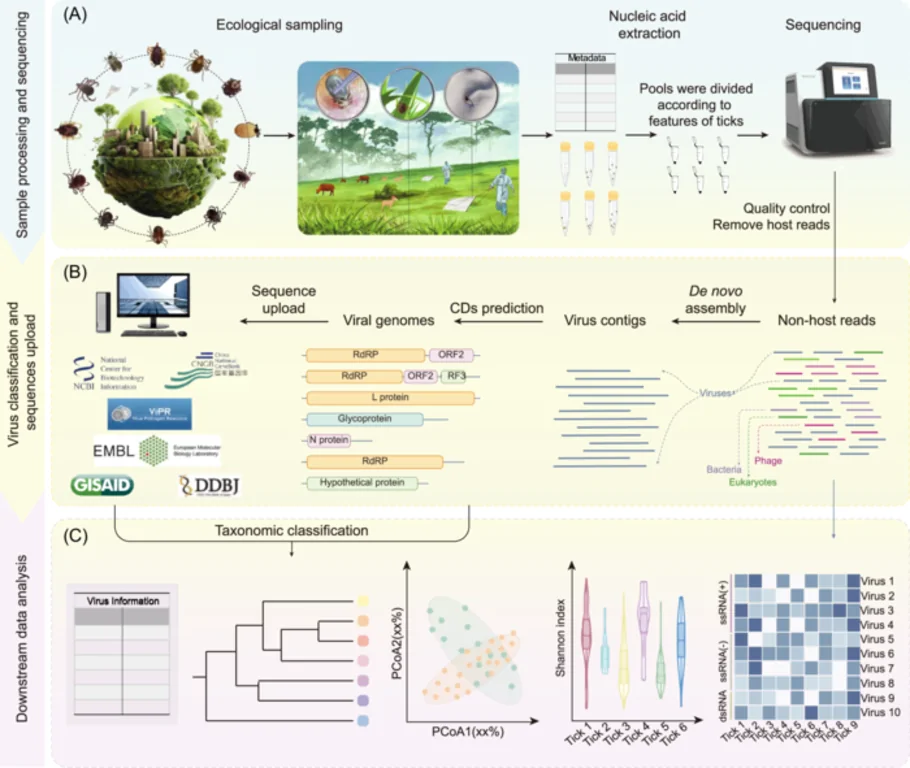
Protocols for the study of tick virome. (A) Sample processing and sequencing, (B) virus classification and sequences upload, and (C) downstream data analysis.

These proposed field survey principles include study design, sampling site selection, sample collection, and preservation to facilitate later meta‐transcriptomic sequencing and analysis (Figure 1A).

### Study design

The sampling locations for field surveys should be determined based on the specific study objectives. Different regions exhibit significant variation in tick viruses, so study design may vary accordingly. However, a study protocol should be established before sample collection. When designing a tick virome study, key factors to consider include the selected study areas, potential tick populations, tick species diversity, ecological diversity of the study region, representativeness of sampling sites, and the scheduling of sites for different sampling locations, and temporary storage of samples collected from different sites.

### Sampling site selection

Ticks primarily inhabit open natural environments such as forests, shrublands, grasslands, and semi‐desert regions. The distribution of different tick species is closely related to factors such as climate, soil, vegetation, and host diversity. For example, *Ixodes persulcatus* is common in high‐latitude mixed coniferous‐broadleaf forests [6], while *Hyalomma asiaticum* habitats in the western edge of the Taklamakan Desert, the Turpan Basin, and the Junggar Basin [7]. It is crucial to identify the local ecological fauna and employ stratified sampling to cover diverse areas. After determining the sampling sites, it is essential to consult literature or conduct field surveys to identify the most suitable seasons for tick presence to ensure sample collection.

Tick sampling, preservation, and metadata documentation (Table S1) are suggested following standardized protocols detailed in Supporting Information Text S2.

### Proposed sample processing and sequencing approaches

In tick virome studies, sample processing and library sequencing construction are essential preparatory steps to ensure sequencing success and data validity. Appropriate sample processing methods can significantly enhance the accuracy and sensitivity of viral detection, establishing a reliable foundation for subsequent analyses. This process involves selecting suitable pooling strategies, preparing libraries, and recording detailed metadata to ensure reproducibility and comparability of the results (Figure 1B). Key aspects such as nucleic acid extraction, library quality control, and sequencing depth must be carefully designed and executed to maximize the capture of viruses. The detailed methods are provided in the Supporting Information Text S3.

### Proposed virome analysis pipeline

Effective data analysis enables researchers to gain insights into viral diversity, distribution, and potential public health risks. This process encompasses multiple aspects, such as virome composition and diversity analysis, sequence assembly and alignment, viral classification, handling contamination and false positives, and fostering data sharing and collaboration (Figure 1C, Text S4). Finally, data sharing through public platforms ensures transparency and fosters collaboration. These analytical steps can not only deepen the scope of research but also provide a robust foundation for further scientific exploration.

### Implementation points

In implementing the above protocol, it is essential to incorporate key elements such as Flexible Operation, Reporting and Communication, and Team Composition and Competency Requirements (Text S5).

## 2 CHALLENGES

**FIGURE 2:**
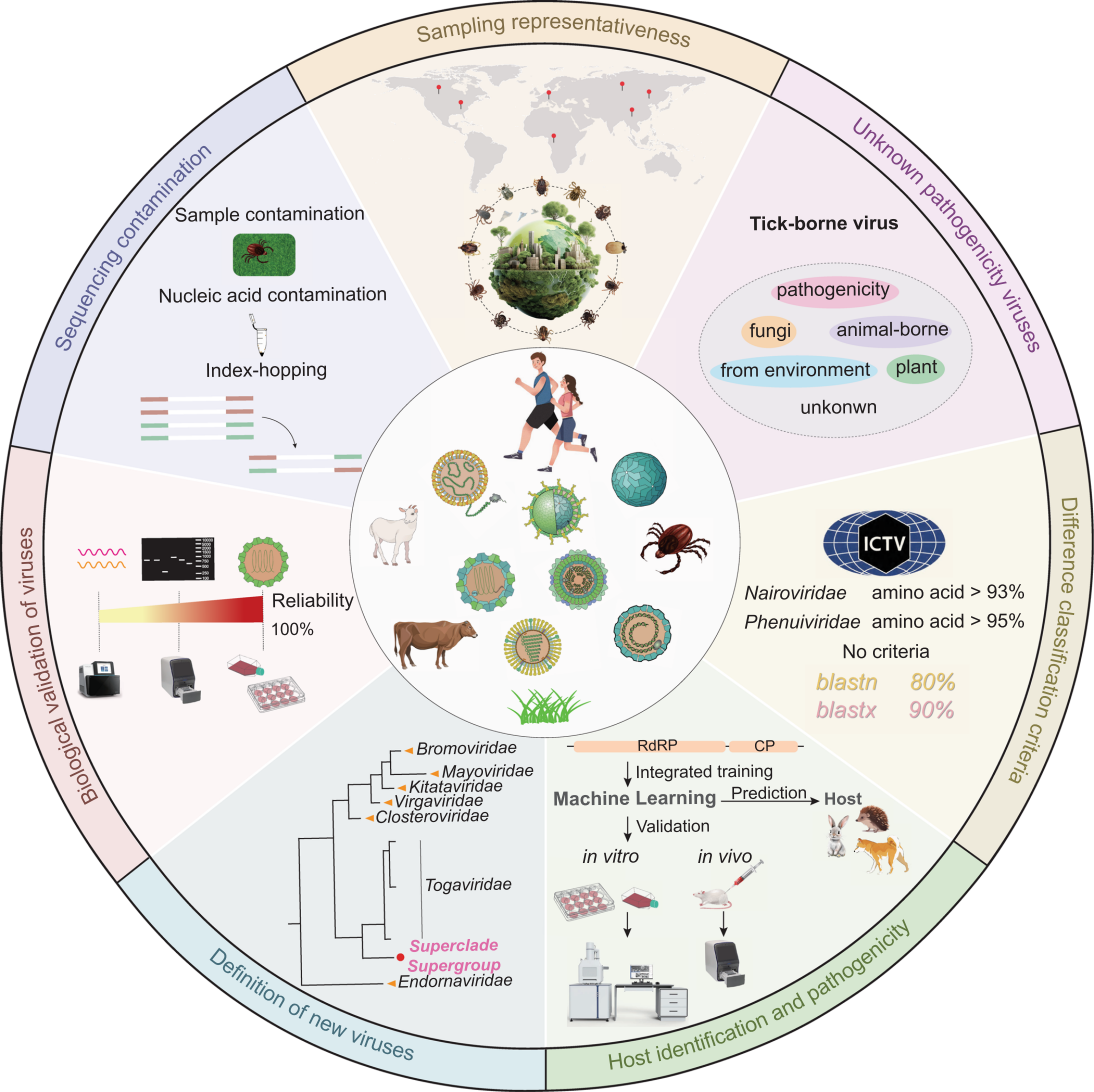
Future challenges for the tick virome studies.

Based on the current research findings, the tick virome reveals the multidimensional and complex relationships among viruses, ticks, hosts, and the environment. The distribution of the tick virome is shaped by tick genetics, host diversity, and ecological complexity [8], suggesting its important role in tick and tick‐borne disease prevention and control. With the standardized methods and integrated data resources, we will be able to conduct more comprehensive studies on the tick virome in the future. However, this field still faces numerous challenges while also presenting rich opportunities (Figure 2).

### Sampling representativeness

The ecological distribution of tick species varies significantly across regions due to differences in climate, host diversity, and environmental complexity. While flexible sampling strategies improve coverage of tick habitats, balancing standardization with adaptability remains challenging. Pooled sequencing of ticks with shared species, gender, and life stage enhances cost‐efficiency but lacks systematic validation for reflecting true viral diversity (Text S6).

### Sequencing contamination

Contamination from reagents, laboratory environments, or index‐hopping in high‐throughput sequencing introduces false positives and quantitative biases. Rigorous mitigation strategies include viral genome integrity and authenticity checks (conserved domains, RT‐PCR/Sanger sequencing) and negative controls (sterile water, reagent blanks), though scalable solutions for large studies remain limited (Text S7).

### Relative high proportion of viruses with unknown pathogenicity

Many tick‐associated viruses originate ambiguously, including from environmental, plant, fungal, or host sources, and their pathogenicity is often unconfirmed. Even animal‐borne viruses such as Tacheng tick virus 1, Nairobi sheep virus, may exhibit delayed zoonotic potential. Continuous monitoring and experimental validation are critical to assess transmission risks (Text S8).

### Biological validation of viruses identified by meta‐transcriptomic sequencing

Viral sequences from meta‐transcriptomics may not confirm biological activity. Virus isolation and culture are important methods to address this issue, but most tick‐borne viruses are difficult to isolate using traditional viral culture techniques. To distinguish whether a virus in a tick is biologically active or capable of causing an infection when a live virus cannot be isolated, three approaches can be used, including fluorescence in situ hybridization for tissue localization, immunofluorescence assays for protein detection, and experimental transmission to SPF animals [9] (Text S9).

### Difference in classification criteria among viral families

Viral classification criteria differ significantly across families, complicating tick virome studies. While ICTV provides conserved gene homology thresholds, genus/species demarcation varies: *Nairoviridae* (<93% RdRP identity), *Phenuiviridae* (<95% RdRP homology). For unclassified viruses (e.g., *Flaviviridae*), provisional thresholds (<90% amino acid similarity for RdRP or <80% nucleotide similarity for full‐genome) are proposed [8, 10]. Dynamic adjustments based on genetic divergence and global collaboration are critical for taxonomic harmonization (Text S10).

### Definition of new viruses

The broad‐scale virome has revealed many previously undefined viruses, including viral dark matter [11], bridging the gaps in viral evolution and expanding the scope of virus classification. These viruses, which transcend the boundaries of existing viral orders and families, are often not classified within the current virus taxonomy system. We refer to them as *superclades* or *supergroups*. The lack of evidence and data regarding their biological characteristics hinders classification. Despite significantly expanding our knowledge of tick‐borne viruses, their biological status and appropriate classification still require further exploration and validation (Text S11).

### Host identification and pathogenicity determination of viruses

Host‐pathogen links and pathogenicity assessment demand integration of sequencing, machine learning [12], and experimental validation including viral isolation and animal models. The most direct evidence remains the confirmation of viral infections in animal population or humans. Active surveillance in virus‐positive regions is essential to confirm natural foci and zoonotic risks (Text S12).

## 3 PERSPECTIVES

### Tick virome will accelerate the discovery and tracing of emerging tick‐borne viruses

A standardized tick virome pipeline enables proactive detection and evolutionary tracing of emerging viruses. Large‐scale meta‐transcriptomics refines viral classification and informs public health alerts [13]. Bioinformatics tools reveal genetic origins and host adaptation mechanisms, guiding targeted surveillance in virus‐positive regions to map transmission routes and hosts [13, 14]. This integrated approach enhances preparedness for emerging tick‐borne threats (Text S13).

### Tick virome will provide fundamental data for the mechanistic study of tick‐borne viruses

Studying the tick virome offers foundational insights into the biological characteristics and pathogenic mechanisms of tick‐borne viruses. Discoveries from virome research encompassing both known and novel viruses, guide their isolation and characterization. Comparative analysis across tick species, developmental stages, and feeding states sheds light on ticks' roles in viral transmission and reveals key clues regarding intra‐tick and tick‐host pathways. For instance, assessing transmission during the ovum period or via blood‐feeding informs our understanding of viral dynamics and ecological distribution. Moreover, insights into virus–host receptor interactions are vital for exploring viral infectivity and pathogenesis. These data are critical for designing targeted interventions and developing effective therapeutic strategies.

### Tick virome will promote studies on virus–tick–host interactions

Large‐scale tick virome data provide evidence to explore infection mechanisms across tick species and interactions among viruses, ticks, and hosts. Understanding virus–tick interactions reveals how virus replication relates to tick genetics, clarifying ticks' role as vectors. Virus–virus interactions, seen in co‐infections, affect viral distribution and transmission [15]. Meanwhile, virus–host interactions determine successful transmission by revealing how viruses engage with the host immune system or receptors, informing on infectivity and pathogenic mechanisms [16]. Such studies offer fresh insights and strategies for preventing tick‐borne diseases.

### Tick virome will improve alerts for high‐risk areas

Identifying the distribution of pathogenic viruses within the tick virome enhances surveillance efforts in high‐risk areas. Monitoring tick‐infested regions has already led to early detection of human infections [13, 14]. By integrating environmental, climatic, and ecological data with tick habitat suitability models, researchers can effectively predict geographical distribution and transmission trends [6]. These integrative surveillance methods support proactive public health responses, enabling early warnings and improved control strategies. Such approaches optimize resource allocation in outbreak scenarios by identifying potential spillover risks, even in regions where cases have not yet been reported.

### Tick virome will enhance prediction for spillover transmission and pathogenicity of tick‐borne viruses

Advancements in artificial intelligence (AI), particularly machine learning (ML) and deep learning (DL), are transforming virus research. In tick virome studies, ML approaches enhance virus discovery, structural prediction, host identification, and transmission modeling [17]. Tools like AlphaFold and ESM‐Fold are revolutionizing viral protein structure prediction by identifying key molecular targets in host interactions [18]. ML‐driven models, trained on multi‐omics data, can forecast cross‐species transmission and overcome host barriers, providing vital insights into viral adaptability and potential human infectivity [12, 19]. Furthermore, integrating these predictive models with experimental validation will refine risk assessments and improve early intervention measures, strengthening our overall capacity to predict spillover and pathogenicity (Text S14).

### Data sharing will facilitate interdisciplinary collaboration

Establishing a standardized data storage and sharing platform for tick virome research will support global collaboration and resource integration. Sharing viral genomic sequences, ecological distribution data, and analytical results will enhance data comparability and advance our collective understanding of tick‐borne viruses. This data sharing not only enhances the efficiency of global research on the tick virome but also provides tick researchers with more comprehensive information on tick virus lineages, distribution, and transmission mechanisms, offering important evidence for the prediction and control of tick‐borne infectious diseases. Strengthening interdisciplinary partnerships across vector biology, epidemiology, virology, and bioinformatics will deepen insights into virus transmission mechanisms and host‐virus‐environment interactions [20]. Through such collaborative efforts, the prevention and control of tick‐borne viruses, as well as fundamental research, will receive more support and momentum.

## AUTHOR CONTRIBUTIONS

**Run‐Ze Ye**: Writing—original draft; methodology; visualization. **Nuo Cheng**: Writing—original draft; methodology; visualization. **Yu‐Yu Li**: Investigation; methodology; visualization. **Ning Wang**: Methodology. **Xiao‐Yang Wang**: Methodology. **Biao Deng**: Methodology; visualization. **Yuguo Chen**: Investigation; supervision. **Li‐Li Ren**: Investigation; supervision. **Wu‐Chun Cao**: Writing—review & editing; supervision; project administration; funding acquisition.

## CONFLICT OF INTEREST STATEMENT

The authors declare no conflicts of interest.

## ETHICS STATEMENT

No animals or humans were involved in this study. This statement confirms that no ethical approval was required for this study.

## Supporting information

Supporting Information 1.

Supporting Information 2.

Supporting Information 3.

## Data Availability

All used data are available in the Supporting Information. The metadata templates have been available and can be obtained from Supporting Information 3. Supplementary materials (text, tables, graphical abstract, slides, videos, Chinese translated version, and update materials) may be found in the online DOI or iMeta Science http://www.imeta.science/imetaomics/. The data that supports the findings of this study are available in the supplementary material of this article.
