# Supplementary material for: Towards tick virome and emerging tick‐borne viruses: Protocols, challenges and perspectives
Source: IMetaOmics. 2025 May 12;2(2):e70022. doi: 10.1002/imo2.70022 (PMC12806352; doi:10.1002/imo2.70022)
Supplement: Supplementary file 1 — Supporting Information 1. [file IMO2-2-e70022-s003.docx]

**Supporting information to:**

**Towards tick virome and emerging tick-borne viruses: protocols, challenges and perspectives**

Run-Ze Ye^1,2#^, Nuo Cheng^2,3,4#^, Yu-Yu Li^2,3,4#^, Ning Wang^2,5^, Xiao-Yang Wang^2,5^, Biao Deng^2^, Yuguo Chen^1^*, Li-Li Ren^3^*, Wu-Chun Cao^2,4,5^*

^1^ Qilu Hospital of Shandong University, Jinan 250012, Shandong, China.

^2^ State Key Laboratory of Pathogen and Biosecurity, Beijing Institute of Microbiology and Epidemiology 100071, Beijing, China.

^3^ Institute of Pathogen Biology, Chinese Academy of Medical Sciences & Peking Union Medical College 102629, Beijing, China.

^4^ Research Unit of Discovery and Tracing of Natural Focus Diseases, Chinese Academy of Medical Sciences 100730, Beijing, China.

^5^ Institute of EcoHealth, School of Public Health, Cheeloo College of Medicine, Shandong University, Jinan 250012, Shandong, China.

^#^ These authors contributed equally: Run-Ze Ye, Nuo Cheng, and Yu-Yu Li

* Corresponding: chen919085@sdu.edu.cn (Yuguo Chen), renliliipb@163.com (Li-Li Ren), caowuchun@126.com (Wu-Chun Cao)

**Supporting Information**

[Text S1. Overview of Current Tick Virome Studies 1](#_Toc194093606)

[Figure S1. Number of tick virome publications and tick-associated virus sequences by year from 2013 to 2024 2](#_Toc194093607)

[Text S2. Sample Collection and Preservation Protocols 3](#_Toc194093608)

[Text S3. Suitable Pooling Strategies, Preparing Libraries, and Recording Detailed Metadata 5](#_Toc194093609)

[Text S4. Data Analysis of Tick Virome 7](#_Toc194093610)

[Text S5. Implementation Points 10](#_Toc194093611)

[Text S6. Sampling Representativeness 11](#_Toc194093612)

[Text S7. Sequencing Contamination 12](#_Toc194093613)

[Text S8. Relative High Proportion of Viruses with Unknown Pathogenicity 13](#_Toc194093614)

[Text S9. Biological Validation of Viruses Identified by Meta-transcriptomic Sequencing 14](#_Toc194093615)

[Text S10. Difference in Classification Criteria among Viral Families 15](#_Toc194093616)

[Text S11. Definition of New Viruses 17](#_Toc194093617)

[Text S12. Host Identification and Pathogenicity Determination of Viruses 18](#_Toc194093618)

[Text S13. Tick Virome Will Accelerate the Discovery and Tracing of Emerging Tick-borne Viruses 19](#_Toc194093619)

[Text S14. Tick Virome Will Enhance Prediction for Spillover Transmission and Pathogenicity of Tick-borne Viruses 20](#_Toc194093620)

[Reference 21](#_Toc194093621)

**Text S1. Overview of Current Tick Virome Studies**

Next-generation sequencing (NGS)-based virome has become a powerful tool for enhancing virus detection and discovery in vectors like ticks [1]. As of October 1, 2024, a literature search using the keywords “tick” and “virus” in PubMed and Web of Science yielded a total of 10,606 publications. Among them, 110 articles had utilized meta-transcriptomic sequencing to analyze the virome of ticks from 30 different countries. The earliest publication appeared in 2013 [2], with a peak in 2021 and 2023, when reports on tick-related virome reached as high as 20 publications per year. The discovery of tick-borne viruses has been accelerating, and the availability of viral sequences in public databases has been rapidly increasing. As of October 1, 2024, there are 17,129 tick-related viral sequences in GenBank, of which 6,737 sequences are from virome studies. The number of sequences available peaked in 2022, accounting for 37.4% (2,520 sequences) of the total sequences related to tick virome, a significant contrast to the single-digit records from 2013 (Figure S1). These sequence databases can serve as foundational resources for scientific research, and meta-transcriptomic sequencing is expected to generate increasingly more viral sequences.

These independent studies on tick-related viruses have produced vast datasets on various tick species, different physiological traits, and diverse geographical distributions. If these datasets adhere to a standardized protocol ensuring the best quality, reliability, and comparability, along with appropriate metadata, the establishment of a centralized data repository would become feasible. To facilitate the creation of a tick virome data repository and promote collaboration and discussions among tick virome researchers, we established the Tick Genome and Microbiome Consortium (TIGMIC), a cooperative group aimed at advancing researches on tick genome, virome and microbiome to shift from passive responses to proactive prevention of tick-borne infections. TIGMIC sequenced 678 samples from six major tick species across mainland China, elucidating the genomic diversity, population genetic structure, and pathogen distribution of these tick species, providing invaluable resources and important theoretical foundations for further research on ticks and tick-borne diseases [3]. Subsequently, meta-transcriptomic sequencing was performed on 31 different tick species from the families Ixodidae and Argasidae, identifying 724 RNA viruses. The 1,801 assembled complete or near-complete viral genomes revealed the extensive diversity in the genomic structures of tick-associated viruses, highlighting ticks as a repository of RNA viruses [4]. Building on these previous findings, TIGMIC further revealed that *Haemaphysalis longicornis* were divided into two clades, and the genomic and ecological characteristics influence the tick virome composition and diversity [5]. Here we call for more research teams interested in tick and tick-borne infections to join TIGMIC for a more extensive global collaboration.

**Figure S1. Number of tick virome publications and tick-associated virus sequences by year from 2013 to 2024**


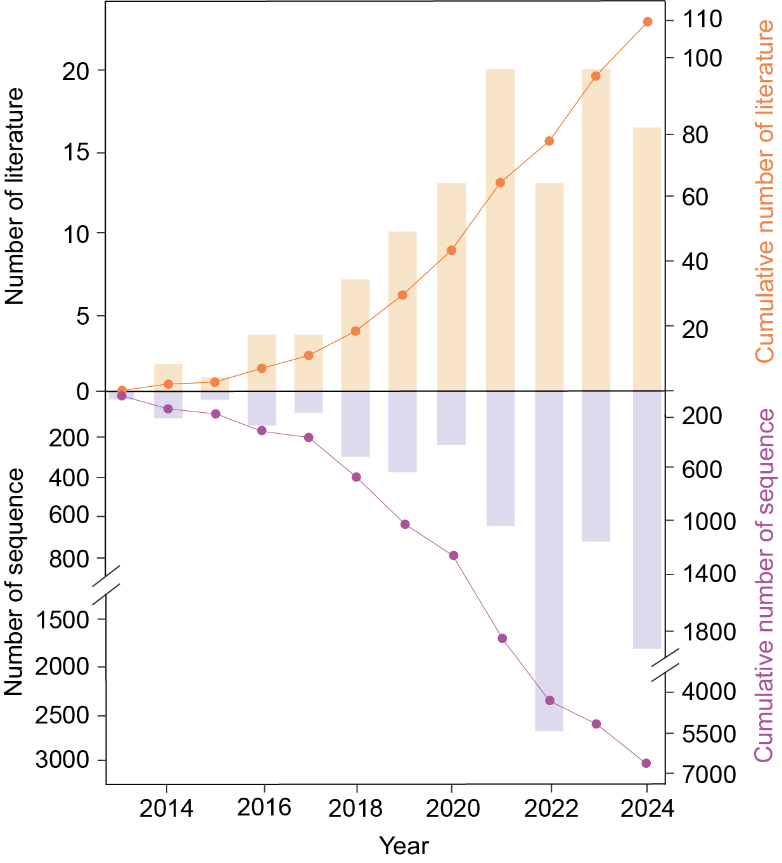


**Text S2. Sample Collection and Preservation Protocols**

Sample Collection

If the study requires the collection of free-living ticks, sampling should be conducted in open, tick-friendly areas where ticks have been previously reported under the guidance of local authorities. The ideal approach is to preserve the ticks’ vitality and appendages, covering the entire sampling area as much as possible. When collecting parasitic ticks, it is important to avoid leaving tick mouthparts inside the host and to maintain the integrity of the ticks.

Currently, three main methods are recommended for sample collection. For free-living ticks, a 1m² white cloth flag can be dragged across the ground [3]. After moving the flag approximately 2-3 meters, it should be lifted and checked for ticks. If ticks are found, they should be removed using tweezers and placed in a centrifuge tube. This process should preserve the ticks’ integrity and vitality. If no ticks are found, the flag should continue to be dragged until ticks are detected. However, if no ticks are found, it might be due to incorrect sweeping technique, or the sampling area may have very few or no free-living ticks. In addition to flagging, free-living ticks can be collected using CO_2_ traps, tick pheromones [6], or host-based attractants to increase tick collection efficiency. For parasitic ticks, field surveys should first be conducted in rural households or livestock areas within the sampling region. Once parasitic ticks are found, tweezers should be used to carefully remove the ticks and place them in prepared centrifuge tubes. Table S1 outlines a list of recommended samples and materials for collection during the early stages of scientific investigation.

Sample Information Recording

After sample collection, it is recommended to use a standardized metadata table designed by TIGMIC to record the information (Table S2). The table consists of two main sections: basic information of ticks and ecological environment of the sampling site. The basic information section should include the tick species, sex, developmental stage, collection source, tick ID, and the number of ticks collected. For collection source which is parasitic tick, it should also include the host species, host ID, and the tick’s feeding status. Tick identification standards can refer to relevant books [7,8] or the recommendations of authoritative ecologists in the field. The ecological environment section should include the country, province (state), city (prefecture), county, and township, longitude, latitude, altitude, collector, collection date and time, and ecological characteristics of the sampling site.

Sample Preservation

For different ticks, there are two primary sample preservation methods before meta-transcriptomic sequencing. For female ticks in a fully engorged state, place them individually in 15 mL centrifuge tubes and allow them to oviposit. The tube opening should be ventilated, and a wet sterile filter paper strip should be placed inside. The centrifuge tube should be placed in a vacuum desiccator with a saturated sulfuric acid solution at the bottom. Petroleum jelly should be applied to the desiccator’s edges to prevent the ticks from escaping. The desiccator should be placed in a climate chamber at 23°C with 90% humidity, alternating 12 hours of light and 12 hours of darkness.

For host-questing and half-engorged ticks, the ticks with the same location, same host, same species, and same gender are placed in the same 15 mL centrifuge tube. The tubes should follow the same process described above, with ventilation and wet sterile filter paper inside the tubes. The centrifuge tubes should be placed in the vacuum desiccator and climate chamber as described.

**Text S3. Suitable Pooling Strategies, Preparing Libraries, and Recording Detailed Metadata**

Sample Processing, Library Construction, and Sequencing

In tick virome research, samples can be processed as individual ticks or pooled ticks for analysis. Individual tick processing allows for more precise viral detection and identification, making it suitable for in-depth studies of specific specimens but often poses challenges due to insufficient nucleic acid quantities. On the other hand, pooling ticks is ideal for initial large-scale screening, improving detection efficiency, especially when sample sizes are large, or resources are limited. For pooling, ticks are typically grouped based on collection location, host association, tick species, life stage, or gender [4,5]. Weighing pooled samples can help ensure sufficient nucleic acid quantities for library preparation.

Following sample processing, library preparation includes steps such as nucleic acid extraction, quality assessment, library construction, and library quality control. Ticks were first rinsed externally with sterile nuclease-free water, then homogenized using a cryogenic grinder or manually pulverized after flash-freezing in liquid nitrogen with a mortar and pestle. Nucleic acids were subsequently extracted using appropriate viral RNA extraction kit. High-throughput sequencing platforms such as Illumina, Nanopore, and PacBio, are widely used, each with distinct advantages depending on research objectives. Illumina platforms provide high accuracy and sequencing depth, making them suitable for detecting low-abundance viruses in complex samples. In contrast, Nanopore and PacBio offer long-read sequencing capabilities, which are advantageous for resolving complex viral genome structures or identifying novel viruses with high genetic divergence [9,10]. Recent studies have demonstrated that combining the precision of short-read sequencing with the extended coverage of long-read sequencing provides a robust solution for meta-transcriptomic assembly, enabling the discovery of significantly more viral sequences compared to single-platform approaches [11−13]. Sequencing platforms should not be viewed as inherently superior or inferior, and their selection depends on specific research needs and technical characteristics. When selecting a platform, researchers should weigh factors such as required read length, cost, and the trade-off between sequencing depth and genome coverage. In tick virome surveys, most field-based investigations focus on pathogen surveillance. Given that viral genomes are relatively compact and cost-effectiveness is a key consideration, Illumina platforms are generally recommended for such studies. Sequencing depth should be optimized to ensure adequate sensitivity for capturing low-abundance viruses, typically ≥10 million reads per library for Illumina-based meta-transcriptome. For studies targeting tick species with large genomes, or blood-sucking ticks, deeper sequencing (e.g., ≥20 million reads) may be necessary to improve detection limits, However, this approach inevitably increases sequencing costs and computational demands for downstream data analysis.

Metadata Documentation

Metadata recording is a crucial component of sample analysis to ensure reproducibility and comparability of results. Based on the initial collection records, metadata should include information such as the tick species, gender, collection site and geographical coordinates, collection time, and other relevant variables for pooled ticks. Additionally, each sequenced library should be annotated with basic information, including the number of ticks per pool, sample ID, laboratory processing methods, sequencing methods, sequencing platform and depth, and details on the storage and labeling of remaining samples. These details facilitate subsequent data analysis and interpretation (see Table S3).

Data Processing

Data quality control involves removing low-quality sequences, primers, and adapter sequences to improve the accuracy of downstream analyses. Various tools have been developed for fastq data preprocessing and quality control, including AfterQC [14], fastp [15], and FastQC [16]. Mitochondrial sequences of ticks obtained through sequencing can be used to construct phylogenetic trees, enabling analyses of genetic relationships among tick populations. Clustering analyses reveal the evolutionary history of different tick species and their ecological roles.

In virome research, removing host associated reads is critical. Tools like bowtie2 [17] and hisat2 [18] can be used to filter out reads that align with the host genome, retaining only viral sequences. Currently, publicly available tick genome data include sequences from 20 tick species (see Table S4).

**Text S4. Data Analysis of Tick Virome**

Virome Composition and Diversity Analysis

Before sequence assembly, virome composition can be analyzed to evaluate viral communities and diversity across different tick species or ticks from diverse geographic regions. Tools like Kraken2 [19] are commonly used to annotate sequencing data, identifying viruses or other microorganisms in samples, and calculating their relative abundances. Combining Kraken2 with tools like Bracken [20] enhances the accuracy of species annotation and provides finer abundance estimates. Diversity analyses, including alpha and beta diversity assessments [21], can then be performed based on relative abundance data to compare viral diversity across sample categories and evaluate ecological, tick species, or other factors influencing viral communities. Common metrics include Shannon and Simpson indexes.

Viral Sequence Assembly

Non-host reads can undergo further assembly, which includes *de novo* assembly or reference-guided assembly. The former reconstructs viral genomes from raw reads, while the latter maps the reads to known viral reference genomes. The choice of assembly strategy depends on sample characteristics and research objectives. Challenges such as low sequence quality, repetitive regions, and assembly errors can arise. Tools like Trinity [22], SPAdes [23], and MEGAHIT [24] are commonly employed, complemented by clean data filtering and quality checks to improve assembly accuracy. Contigs will be formed by assembling multiple reads and can be further analyzed to evaluate sequence completeness, accuracy, and evolutionary relationship.

Virus Taxonomy

The BLASTx and BLASTn [25] are commonly used to compare contigs with public databases such as nt and nr databases from NCBI to determine the taxonomic relationship. Non-viral contigs are filtered out based on above results, and sequences related to bacteriophages or environmental viruses may also be excluded depending on research goals. Open reading frames (ORFs) in viral genomes can be predicted using ORFfinder [26], and annotated coding sequences (CDs) can be compared against established criteria from the International Committee on Taxonomy of Viruses (ICTV). Viral species classification vary among viral families and genera. For viral taxonomy lacking clearly defined standards in ICTV, thresholds of < 90% amino acid similarity for RNA-dependent RNA polymerase (RdRP) or capsid protein (CP), or < 80% nucleotide similarity for full genomes, are often used to provisionally distinguish novel species [27,28]. Annotation accuracy directly influences our understanding of tick viral community structure and phylogenetic characteristics.

Phylogenetic analysis is a key method to investigate the evolution and classification of tick-borne viruses. By constructing phylogenetic trees, researchers can reveal genetic relationships among viral strains, infer transmission patterns, and evaluate public health risks. Common approaches include maximum likelihood (ML), Bayesian inference, and neighbor-joining methods, supported by tools such as MEGA [29], RAxML [30], MrBayes [31], and IQ-TREE [32].

Removing of Contamination and False Positives

Due to the unbiased nature of meta-transcriptomics, both contaminant and biologically relevant viral sequences present in a sample are sequenced simultaneously, making it challenging to distinguish between the two [33]. This challenge is amplified by the vast majority of viruses remaining unclassified and the absence of prior knowledge about the viral taxonomy present in any given sample. Contamination may occur at various stages of sample preparation, including collection, nucleic acid extraction, and library construction and sequencing.

Since virus are often present at significantly lower abundance than host or co-infecting microbial transcripts, increasing sequencing depth is critical for detecting and characterizing low-abundance biologically relevant viruses. However, deeper sequencing also exacerbates contamination issues, as even trace viral sequences in reagents can be detected [34]. Another potential source of contamination is index-hopping, a phenomenon where reads are incorrectly assigned between libraries, leading to cross-contamination [35].

To mitigate false positives and address potential contamination during data analysis, strategies such as including blank controls, performing nucleic acid validation, and verifying sequence abundance can be employed. Additionally, contamination within reference databases poses another challenge. Public databases may contain incorrect viral sequences, which limits their utility in bioinformatic pipelines for viral discovery [33].

Data Sharing

The importance of data sharing within the scientific community cannot be overstated. Platforms such as NCBI [16], CNGBdb, and GISAID provide invaluable resources for researchers to share and access datasets. Upon completing sample analyses, sequences and associated metadata should be uploaded to public databases to promote scientific collaboration and information sharing. Beyond uploading assembled and taxonomically annotated sequences, raw sequencing data, appropriately quality-controlled, should also be made available and securely stored to facilitate reproducibility and traceability. Ensuring open accessibility to data not only supports subsequent research and innovation but also enhances the transparency and impact of scientific investigations. This collaborative approach fosters a unified effort in addressing the complexities of emerging viral threats.

**Text S5. Implementation Points**

Flexible Operation

Research teams can conduct parallel tasks employing different technical approaches, rather than adhering to a strictly linear order. For instance, a study might begin with sample collection from a single tick species, followed by preliminary meta-transcriptomic sequencing to identify potential viruses. Based on initial findings and field conditions, subsequent sampling and analysis can be adjusted, or the investigation focus can shift to different regions or analytical priorities. This adaptability allows researchers to modify strategies in response to real-time findings, enabling more effective responses to potential public health threats.

Reporting and Communication

Timely reporting and integration of investigation findings are essential. Research teams should communicate progress to relevant authorities, including results of tick sample analyses, identified viruses, and evaluations of potential transmission pathways. By synthesizing evidence from various technical components, researchers can provide critical insights for subsequent scientific studies, control measures, and public health decision-making. Sharing this information not only enhances research transparency but also fosters collaboration among diverse research teams.

Team Composition and Competency Requirements

Effective tick virome research requires multidisciplinary specialists with expertise on virology, entomology, molecular biology, genomics, and epidemiology. The complex interactions between ticks and pathogens necessitate a comprehensive understanding of how these factors effect virus transmission. This specificity extends to knowledge of tick biology and ecological behaviors, as well as the impact of human activities on tick habitats and their relationships with hosts. Cross-disciplinary collaboration is essential to address the challenges posed by TBVs and to develop effective public health prevention and control strategies. The capacity for bioinformatics analysis and computational resources should be continuously improved to support these investigations.

**Text S6. Sampling Representativeness**

The ecological distribution characteristics of tick species exhibit significant regional differences [36]. The geographical environmental conditions, host species, and tick species diversity in different regions can all influence the rationality of the sampling approach. This regional variability makes it difficult for a unified sampling method to meet the needs of all research scenarios, resulting in a lack of direct comparability between the results of different studies. Based on the sampling recommendations we proposed, researchers can flexibly design sampling points according to the ecological characteristics of the surveyed area to comprehensively cover the tick ecological distribution in the study region. However, ensuring the standardization and consistency of the survey results while maintaining flexibility remains an important challenge.

In addition, in the sequencing design of samples, the analysis method of pooling ticks with similar characteristics for sequencing is a commonly used approach [37]. Under the condition that the nucleic acid quantity meets the sequencing requirements, this method allows for a cost-effective investigation of the viral burden in ticks in the study area. The nucleic acid quantity in a single tick sample is often insufficient for library construction and sequencing, whereas pooling analysis can provide a broader overall overview. However, there is a lack of systematic evaluation studies on whether pooled sample library construction can accurately reflect the true distribution and composition of viruses. How to ensure data representativeness while meeting sequencing conditions still requires further exploration and optimization of solutions

**Text S7. Sequencing Contamination**

Tick virome research is susceptible to various types of contamination during sample collection, handling, and analysis, including exogenous contamination such as that introduced by reagent kits [34], contamination from the laboratory environment, and cross-contamination between samples. For example, the common issue of index-hopping in high-throughput sequencing [35] can increase the risk of false-positive results and introduce systematic quantitative biases by misassigning reads between samples. In addition, environmental sources of contamination, such as host skin, vegetation, and soil microorganisms, can also interfere with viral detection results. To mitigate these issues, rigorous validation strategies are essential. First, the integrity of viral genomes should be confirmed by assessing the completeness of CDs and conserved functional domains, followed by RT-PCR amplification and Sanger sequencing of the original samples to exclude assembly artifacts. Concurrently, sequencing experiments should incorporate negative controls such as sterile water and extraction reagent blanks to identify technical contaminants, as demonstrated in virome studies of arthropod-associated viruses [38]. Although some techniques have been developed to reduce such issues [33], there is still a lack of universally applicable and cost-effective solutions, especially for large-scale sample analysis. When reporting results, we should be cautious about identifying false positives and consider incorporating control experiments to verify the reliability of the analysis.

**Text S8. Relative High Proportion of Viruses with Unknown Pathogenicity**

A large number of viruses in ticks had been identified, but the pathogenicity of many viruses remains unclear. On one hand, their sources are often ambiguous. Ticks, as blood-feeding arthropods, have a complex life cycle that involves various ecological environments and host types. Therefore, the viruses found in ticks may originate from environmental viruses, fungal viruses, plant viruses, or host animal viruses. Some viruses may merely be residual components ingested by ticks, rather than true tick-borne viruses. Accurately identifying whether these viruses are solely from the environment and whether they can replicate within ticks is a challenge. This not only requires support from viromic data but also demands in-depth studies related to tick physiology and ecological characteristics.

On the other hand, even for animal-borne viruses, their pathogenicity is often unclear. Distinguishing whether these viruses can achieve cross-species transmission from ticks to other animal hosts, or even to humans, cannot be definitively concluded through viromic analysis alone. Further experimental validation is needed to determine their transmission potential and pathogenicity. Although the pathogenicity of these viruses is unknown, their potential public health risks should not be underestimated. Some viruses initially discovered in ticks were not confirmed to be related to human diseases in the early stages, but years later were found to have human infection potential, such as Tacheng tick virus 1 [39], Nairobi sheep virus [40], and Beiji nairovirus [41]. Therefore, for these viruses with unclear public health significance, in addition to comprehensive biological characterization, continuous monitoring of potentially infective viruses in the human population should be conducted. Through early warning and appropriate intervention, the risk of potential pathogens spreading to humans can be avoided, thereby reducing the likelihood of emerging public health events.

**Text S9. Biological Validation of Viruses Identified by Meta-transcriptomic Sequencing**

Whether the assembled sequences obtained through high-throughput sequencing accurately reflect the presence of viruses remains a core challenge in tick virome research. Most current studies rely on sequences assembled from high-throughput sequencing, but it is still unclear whether these sequences represent the virus's activity or its true presence in ticks. Additionally, the detection of viral sequences in ticks does not necessarily indicate that the virus is biologically active or capable of causing infection. Therefore, the lack of biological validation may limit the credibility and scientific significance of these data. Virus isolation and culture are important methods to address this issue, but most tick-borne viruses are difficult to isolate using traditional viral culture techniques [42]. Especially for newly discovered tick-borne viruses, whose biological characteristics are unknown, conventional culture methods or host cell lines may not be suitable for their replication. Tick samples are usually collected from field sites, and once collected, it may be difficult to obtain sufficiently fresh or viable positive samples for isolation experiments, further complicating the pathogen validation process. In cases where virus isolation is limited, using specific primers for PCR (Polymerase Chain Reaction) amplification of the original samples followed by Sanger sequencing to verify high-throughput sequencing results may still be one of the approaches to increase the reliability of the findings. To distinguish whether a virus in a tick is biologically active or capable of causing an infection when a live virus cannot be isolated, three approaches can be used. First, a set of specific probes are designed according to the virus genome sequence and labeled with fluorophores for fluorescence in situ hybridization (FISH) experiments to observe the presence of viral particles in the midgut, salivary glands, or ovaries of ticks [43]. Second, specific antibodies are prepared based on the virus amino acid sequences and used for immunofluorescence assay (IFA) to detect viral infection in ticks. Third, although challenging, an attempt can be made to allow positive ticks to directly bite specific pathogen free (SPF) animals, and detect the same viral sequences in the experimental animals to confirm the presence of live virus.

**Text S10. Difference in Classification Criteria among Viral Families**

The classification criteria for viruses differ between families, posing challenges in viral classification and identification. Especially in tick virome, a large number of novel viruses are often detected, and their classification typically relies on phenotypes inferred from genome analysis and sequence relatedness inferred from phylogeny, homology detection, and divergence metrics [44]. The current species definition formulated by the International Committee on Taxonomy of Viruses (ICTV) adheres to a set of minimum rules outlined in the *International Code of Virus Classification and Nomenclature* (https://ictv.global/report). However, classification criteria can vary within different virus families, such as genome length, amino acid sequence similarity of conserved genes, host range, or evolutionary relationships. These differences increase the complexity of viral classification and identification.

In particular, many tick-borne viruses have not yet been included in the ICTV classification system. One commonly used criterion for classification is the homology of conserved proteins **(**<https://ictv.global/>). For example, in the *Nairoviridae* family, an RdRP sequence identity of <93% represents different species [45], in the *Phenuiviridae* family, species distinction is based on a homology of less than 95% in the amino acid sequence of the RdRP [46]. Although the criteria for genus classification vary across families, there are some similarities at the species level. For instance, in the *Botourmiaviridae* family, members of different genera are considered the same genus if the full conserved region of the RdRP protein has greater than 70% similarity, and the same species if it exceeds 90% similarity [47], in the *Chuviridae* family, viruses from different genera should have less than 31% homology in the conserved L protein, and different species should have less than 90% homology in the conserved L protein sequence [48], in the *Iflaviridae* family, the amino acid sequence of the capsid protein for the same virus should have more than 90% homology [49].

For many other families that do not have clearly defined classification standards, such as *Flaviviridae* and *Dicistroviridae*, we suggest a provisional threshold: a standard of <90% amino acid sequence homology for RdRP or CP and <80% nucleotide sequence homology of complete viral genome for defining a new member of virus in a certain viral genus [4,5]. In fact, as the continuous discovery of viruses expands our understanding of virus diversity, the classification criteria can be dynamically adjusted based on genetic and evolutionary relationship. For instance, with the increasing number of viruses previously defined as toti-like viruses, they do not cluster together in the phylogenetic tree. Consequently, the ICTV has now classified these viruses into different families within the order *Ghabrivirales*. In the face of these challenges, it is recommended that researchers combine the existing ICTV classification standards while also considering the phylogenetic characteristics and genetic distance of tick-borne viruses to improve classification approach suitable for virus taxonomy. More important, international collaboration and information sharing are crucial for unifying and optimizing the tick virus classification system, which will lay a more solid foundation for the identification and naming of new viruses.

**Text S11. Definition of New Viruses**

The broad-scale virome has revealed many previously undefined viruses, including viral dark matter [50], bridging the gaps in viral evolution and expanding the scope of virus classification. These viruses, which transcend the boundaries of existing viral orders and families, are often not classified within the current virus taxonomy system. We refer to them as superclades or supergroups [51]. They cannot be placed in any of the established viral orders or families, or they fall between two orders or two families. These viruses were largely overlooked in previous tick virome research, but with the development of sequencing technologies, their numbers have grown, and they now rival the number of species in some known viral orders. The diversity of these viruses has enhanced our understanding of tick-borne viruses, but due to the lack of evidence and data regarding their biological characteristics, it is challenging to fully uncover the genomic features of each virus within a superclade, and we lack a clear classification for them. Their true biological significance and taxonomic position remain unclear. Therefore, whether this naming approach will be truly accepted in the virology community and whether it has broad applicability remains controversial. Despite significantly expanding our knowledge of tick-borne viruses, their biological status and appropriate classification still require further exploration and validation.

**Text S12. Host Identification and Pathogenicity Determination of Viruses**

Last but not least, identifying the host of a virus and determining its pathogenicity are two challenges. High-throughput sequencing combined with bioinformatics methods can facilitate the discovery of new viruses and obtain their full-length genome sequences. Although machine learning and other computational methods have been used to predict virus hosts and vectors based on large-scale existing data [52], prediction analysis alone cannot fully identify the animal host or pathogenicity of a virus. Viruses detected through sequencing must first be successfully isolated and then subjected to in vitro and in vivo experiments to assess their infectivity and pathogenicity. The most direct evidence remains the confirmation of viral infections in animal population or humans. Therefore, it is necessary to strengthen active surveillance of animals and humans in regions where virus-positive ticks are found to determine the natural foci of the virus and assess its pathogenic potential in both animals and humans.

**Text S13. Tick Virome Will Accelerate the Discovery and Tracing of Emerging Tick-borne Viruses**

A standardized tick virome analysis pipeline will provide strong support for the discovery and tracing of emerging tick-borne viruses. As the number of newly emerging tick-borne viruses and tick-borne diseases continue to increase, tick virome research will offer effective methods for the proactive detection of these novel viruses. Through large-scale and unbiased virome analysis, it is possible to further understand the evolutionary processes of different types of viruses and refine the classification of the tick virome. Systematic virome analysis will not only enable the timely identification of unknown tick-borne viruses but also provide data support for public health early warning and control efforts [53], thus improving the response speed to emerging tick-borne infectious diseases.

Furthermore, by utilizing bioinformatics tools for in-depth analysis of the genomes of emerging viruses, we can trace their potential evolutionary origins and provide important clues for understanding the ecological characteristics and transmission chains of tick-borne viruses. By revealing the genetic and evolutionary features of viruses, we can better identify the relationships between viruses and hosts, understand the adaptive evolution processes of emerging tick-borne viruses, and thus provide a basis for the development of control strategies.

Most importantly, after the discovery of emerging tick-borne viruses, we can further guide targeted surveys of host animals and human populations in surrounding areas based on the virus's distribution and ecological characteristics [53,54]. This will not only help further determine the potential transmission routes and sources of the virus but also assist in identifying possible animal hosts or vectors, defining the virus's epidemic characteristics in human populations, and providing scientific evidence for viral traceability.

**Text S14. Tick Virome Will Enhance Prediction for Spillover Transmission and Pathogenicity of Tick-borne Viruses**

With the continuous advancement of artificial intelligence (AI), particularly its subset machine learning (ML), which focuses on data-driven model training, the boundaries of virus research are being constantly expanded. ML, especially deep learning (DL), has emerged as a powerful tool in viral studies, driving revolutionary developments in virus detection, host prediction, and transmission model construction. In tick virome research, ML-based approaches hold unprecedented potential for virus discovery, structural prediction, host identification, and the development of prevention and control strategies. ML-driven metagenomic mining techniques may allow us to explore viral diversity within ticks that has not yet been discovered [62].

In the study of virus structure, deep learning has revolutionized traditional prediction methods. Advanced models such as AlphaFold can predict the three-dimensional structure of viral proteins, thereby helping to identify key molecular targets involved in host interactions. This DL-driven structural biology approach not only unravels the "identity mystery" of viruses but may also help reveal the role of specific host genes in viral adaptation. Researchers have combined phylogenetic analysis with ML-based structure prediction, tools such as AlphaFold2 and ESM-Fold to predict the structure of *Flavivirus* glycoproteins and employed Foldseek for structural homology searches [63]. The application of ML in this field will significantly enhance our ability to predict virus cross-species transmission and host adaptability, especially for tick-borne viruses [52].

ML models, especially deep learning architectures have shown great potential in virus-host prediction. Trained on multi-omics datasets, they can also predict whether a virus can overcome host barriers, transmit across species, and even assess its potential to infect humans [64]. By analyzing the interactions between tick-borne viruses and hosts, ML frameworks help identify the potential role of different animal species in virus transmission. This capability is especially valuable for studying viruses whose hosts cannot be directly identified through traditional experimental methods. Looking ahead, the integration of ML with multi-omics data and experimental validation will further refine predictive models.

**Reference**

1. Ko, Karrie K. K., Kern Rei Chng, and Niranjan Nagarajan. 2022. “Metagenomics-enabled microbial surveillance.” *Nature Microbiology* 7: 486-496. https://doi.org/10.1038/s41564-022-01089-w
2. Tokarz, Rafal, Stephen Sameroff, Maria Sanchez Leon, Komal Jain, and W. Ian Lipkin. 2014. “Genome characterization of Long Island tick *rhabdovirus*, a new virus identified in *Amblyomma americanum* ticks.” *Virology Journal* 11: 26. https://doi.org/10.1186/1743-422X-11-26
3. Jia, Na, Jinfeng Wang, Wenqiang Shi, Lifeng Du, Yi Sun, Wei Zhan, Jia-Fu Jiang, et al. 2020. “Large-scale comparative analyses of tick genomes elucidate their genetic diversity and vector capacities.” *Cell* 182: 1328-1340.e13. https://doi.org/10.1016/j.cell.2020.07.023
4. Ni, Xue-Bing, Xiao-Ming Cui, Jin-Yue Liu, Run-Ze Ye, Yu-Qian Wu, Jia-Fu Jiang, Yi Sun, et al. 2023. “Metavirome of 31 tick species provides a compendium of 1,801 RNA virus genomes.” *Nature Microbiology* 8: 162-173. https://doi.org/10.1038/s41564-022-01275-w
5. Ye, Run-Ze, Yu-Yu Li, Da-Li Xu, Bai-Hui Wang, Xiao-Yang Wang, Ming-Zhu Zhang, Ning Wang, et al. 2024. “Virome diversity shaped by genetic evolution and ecological landscape of *Haemaphysalis longicornis*.” *Microbiome* 12: 35. https://doi.org/10.1186/s40168-024-01753-9
6. Barré, N., G. I. Garris, and O. Lorvelec. 1997. “Field sampling of the tick *Amblyomma variegatum* (Acari: *Ixodidae*) on pastures in Guadeloupe; attraction of CO_2_ and/or tick pheromones and conditions of use.” *Experimental & Applied Acarology* 21:95-108. https://doi.org/10.1023/b:appa.0000031788.88306.77
7. Sun, Yi, and Rongman Xu. 2016. “Fauna Sinica lnvertebrate，Arachnida.” *lxodida Science Press*, Beijing, In press.
8. Barker, S. C., and A. Murrell. 2004. “Systematics and evolution of ticks with a list of valid genus and species names.” *Parasitology* 129Suppl: S15-S36. https://doi.org/10.1017/s0031182004005207
9. Quick, Joshua, Nicholas J. Loman, Sophie Duraffour, Jared T. Simpson, Ettore Severi, Lauren Cowley, Joseph Akoi Bore, et al. 2016. “Real-time, portable genome sequencing for Ebola surveillance.” *Nature* 530: 228-232. https://doi.org/10.1038/nature16996
10. Hall, Michael B., Marie Sylvianne Rabodoarivelo, Anastasia Koch, Anzaan Dippenaar, Sophie George, Melanie Grobbelaar, Robin Warren, et al. 2023. “Evaluation of Nanopore sequencing for Mycobacterium tuberculosis drug susceptibility testing and outbreak investigation: a genomic analysis.” *The Lancet Microbe* 4: e84-e92. https://doi.org/10.1016/S2666-5247(22)00301-9
11. Wang, Huarui, Chuqing Sun, Yun Li, Jingchao Chen, Xing-Ming Zhao, and Wei-Hua Chen. 2024. “Complementary insights into gut viral genomes: a comparative benchmark of short- and long-read metagenomes using diverse assemblers and binners.” *Microbiome* 12: 260. https://doi.org/10.1186/s40168-024-01981-z
12. Chen, Jingchao, Chuqing Sun, Yanqi Dong, Menglu Jin, Senying Lai, Longhao Jia, Xueyang Zhao, et al. 2024. “Efficient recovery of complete gut viral genomes by combined short-and long-read sequencing.” *Advanced Science* 11: e2305818. https://doi.org/10.1002/advs.202305818
13. Cook, Ryan, Andrea Telatin, Shen-Yuan Hsieh, Fiona Newberry, Mohammad A. Tariq, Dave J. Baker, Simon R. Carding, and Evelien M. Adriaenssens. 2024. “Nanopore and Illumina sequencing reveal different viral populations from human gut samples.” *Microbial Genomics* 10: 001236. https://doi.org/10.1099/mgen.0.001236
14. Chen, Shifu, Tanxiao Huang, Yanqing Zhou, Yue Han, Mingyan Xu, and Jia Gu. 2017. “AfterQC: automatic filtering, trimming, error removing and quality control for fastq data.” *BMC Bioinformatics* 18(Suppl 3): 80. https://doi.org/10.1186/s12859-017-1469-3
15. Chen, Shifu, Yanqing Zhou, Yaru Chen, and Jia Gu. 2018. “fastp: an ultra-fast all-in-one FASTQ preprocessor.” *Bioinformatics* 34: i884-i890. https://doi.org/10.1093/bioinformatics/bty560
16. Brown, Joseph, Meg Pirrung, and Lee Ann McCue.2017. “FQC Dashboard: integrates FastQC results into a web-based, interactive, and extensible FASTQ quality control tool.” *Bioinformatics* 33: 3137-3139. https://doi.org/10.1093/bioinformatics/btx373
17. Langmead, Ben, and Steven L. Salzberg. 2012. “Fast gapped-read alignment with Bowtie 2.” *Nature Methods* 9: 357-359. https://doi.org/10.1038/nmeth.1923
18. Kim, Daehwan, Joseph M. Paggi, Chanhee Park, Christopher Bennett, and Steven L Salzberg. 2019. “Graph-based genome alignment and genotyping with HISAT2 and HISAT-genotype.” *Nature Biotechnology* 37: 907-915. https://doi.org/10.1038/s41587-019-0201-4
19. Wood, Derrick E, Jennifer Lu, and Ben Langmead. 2019. “Improved metagenomic analysis with Kraken 2.” *Genome Biology* 20: 257. https://doi.org/10.1186/s13059-019-1891-0
20. Lu, Jennifer, Florian P. Breitwieser, Peter Thielen, and Steven L. Salzberg. 2017. “Bracken: estimating species abundance in metagenomics data.” *PeerJ Computer Science* 3: e104. https://doi.org/10.7717/peerj-cs.104
21. Lagkouvardos, Ilias, Sandra Fischer, Neeraj Kumar, and Thomas Clavel. 2017. “Rhea: a transparent and modular R pipeline for microbial profiling based on 16S rRNA gene amplicons.” *PeerJ* 5: e2836. https://doi.org/10.7717/peerj.2836
22. Haas, Brian J., Alexie Papanicolaou, Moran Yassour, Manfred Grabherr, Philip D. Blood, Joshua Bowden, Matthew Brian Couger, et al. 2013. “*De novo* transcript sequence reconstruction from RNA-seq using the Trinity platform for reference generation and analysis.” *Nature Protocols* 8: 1494-1512. https://doi.org/10.1038/nprot.2013.084
23. Bankevich, Anton, Sergey Nurk, Dmitry Antipov, Alexey A. Gurevich, Mikhail Dvorkin, Alexander S. Kulikov, Valery M. Lesin, et al. 2012. “SPAdes: a new genome assembly algorithm and its applications to single-cell sequencing.” *Journal of Computational Biology* 19: 455-477. https://doi.org/10.1089/cmb.2012.0021
24. Li, Dinghua, Chi-Man Liu, Ruibang Luo, Kunihiko Sadakane, and Tak-Wah Lam. 2015. “MEGAHIT: an ultra-fast single-node solution for large and complex metagenomics assembly via succinct de Bruijn graph.” *Bioinformatics* 31: 1674-1676. https://doi.org/10.1093/bioinformatics/btv033
25. Camacho, Christiam, George Coulouris, Vahram Avagyan, Ning Ma, Jason Papadopoulos, Kevin Bealer, and Thomas L Madden. 2009. “BLAST+: architecture and applications.” *BMC Bioinformatics* 10: 421. https://doi.org/10.1186/1471-2105-10-421
26. Sayers, Eric W., Evan E. Bolton, J. Rodney Brister, Kathi Canese, Jessica Chan, Donald C. Comeau, Ryan Connor, et al. 2011. “Database resources of the National Center for Biotechnology Information.” *Nucleic Acids Research* 39(Database issue): D38-D51. https://doi.org/10.1093/nar/gkab1112
27. Shi, Mang, Xian-Dan Lin, Jun-Hua Tian, Liang-Jun Chen, Xiao Chen, Ci-Xiu Li, Xin-Cheng Qin, et al. 2016. “Redefining the invertebrate RNA virosphere.” *Nature* 540: 539-543. https://doi.org/10.1038/nature20167
28. Shi, Wenqiang, Mang Shi, Teng-Cheng Que, Xiao-Ming Cui, Run-Ze Ye, Luo-Yuan Xia, Xin Hou, et al. 2022. “Trafficked Malayan pangolins contain viral pathogens of humans.” *Nature Microbiology* 7: 1259-1269. https://doi.org/10.1038/s41564-022-01181-1
29. Kumar, Sudhir, Glen Stecher, Michael Li, Christina Knyaz, and Koichiro Tamura. 2018. “MEGA X: Molecular evolutionary genetics analysis across computing platforms.” *Molecular Biology and Evolution* 35: 1547-1549. https://doi.org/10.1093/molbev/msy096
30. Stamatakis, Alexandros. 2014. “RAxML version 8: a tool for phylogenetic analysis and post-analysis of large phylogenies.” *Bioinformatics* 30: 1312-1313. https://doi.org/10.1093/bioinformatics/btu033
31. Pang, Shuai, Rebecca J. Stones, Ming-Ming Ren, Xiao-Guang Liu, Gang Wang, Hong-ju Xia, Hao-Yang Wu, Yang Liu, and Qiang Xie. 2015. “GPU MrBayes V3.1: MrBayes on graphics processing units for protein sequence data.” *Molecular Biology and Evolution* 32: 2496-2497. https://doi.org/10.1093/molbev/msv129
32. Nguyen, Lam-Tung, Heiko A. Schmidt, Arndt von Haeseler, and Bui Quang Minh. 2015. “IQ-TREE: a fast and effective stochastic algorithm for estimating maximum-likelihood phylogenies.” *Molecular Biology and Evolution* 32: 268-274.
33. Cobbin, Joanna Ca, Justine Charon, Erin Harvey, Edward C. Holmes, and Jackie E. Mahar. 2021. “Current challenges to virus discovery by meta-transcriptomics.” *Current Opinion in Virology* 51: 48-55. https://doi.org/10.1016/j.coviro.2021.09.007
34. Asplund, M., K. R. Kjartansdóttir, S. Mollerup, L. Vinner, H. Fridholm, J. A. R. Herrera, J. Friis-Nielsen, et al. 2019. “Contaminating viral sequences in high-throughput sequencing viromics: a linkage study of 700 sequencing libraries.” *Clinical Microbiology and Infection* 25: 1277-1285. https://doi.org/10.1016/j.cmi.2019.04.028
35. MacConaill, Laura E., Robert T. Burns, Anwesha Nag, Haley A. Coleman, Michael K. Slevin, Kristina Giorda, Madelyn Light, et al. 2018. “Unique, dual-indexed sequencing adapters with UMIs effectively eliminate index cross-talk and significantly improve sensitivity of massively parallel sequencing.” *BMC Genomics* 19: 30. https://doi.org/10.1186/s12864-017-4428-5
36. Ma, Rui, Chunfu Li, Ai Gao, Na Jiang, Jian Li, Wei Hu, and Xinyu Feng. 2024. “Tick species diversity and potential distribution alternation of dominant ticks under different climate scenarios in Xinjiang, China.” *PLoS Neglected Tropical Diseases* 18: e0012108. https://doi.org/10.1371/journal.pntd.0012108
37. Anand, Santosh, Eleonora Mangano, Nadia Barizzone, Roberta Bordoni, Melissa Sorosina, Ferdinando Clarelli, Lucia Corrado, Filippo Martinelli Boneschi, Sandra D'Alfonso, and Gianluca De Bellis. 2016. “Next generation sequencing of pooled samples: guideline for variants' filtering.” *Scientific Reports* 6: 33735. https://doi.org/10.1038/srep33735
38. Mahar, Jackie E., Mang Shi, Robyn N. Hall, Tanja Strive, and Edward C. Holmes. 2020. “Comparative Analysis of RNA Virome Composition in Rabbits and Associated Ectoparasites.” *Journal of Virology* 94: e02119-19. https://doi.org/10.1128/JVI.02119-19
39. Liu, Xiafei, Xu Zhang, Zedong Wang, Zhihui Dong, Songsong Xie, Mengmeng Jiang, Ruixia Song, et,al. 2020. “A tentative Tamdy orthonairovirus related to febrile illness in northwestern China.” *Clinical Infectious Diseases* 70: 2155-2160. https://doi.org/10.1093/cid/ciz602
40. Dandawate, C. N., and K. V. Shah. 1969. “Ganjam virus: a new arbovirus isolated from ticks *Haemaphysalis* intermedia Warburton and Nuttall, 1909 in Orissa, India.” *Indian Journal of Medical Research* 57: 799-804.
41. Wang, Yan-Chun, Zhengkai Wei, Xiaolong Lv, Shuzheng Han, Zedong Wang, Changfa Fan, Xu Zhang, et al. 2021. “A new nairo-like virus associated with human febrile illness in China.” *Emerging Microbes & Infections* 10: 1200-1208. https://doi.org/10.1080/22221751.2021.1936197
42. Kobayashi, Daisuke, Yusuke Inoue, Ryosuke Suzuki, Mami Matsuda, Hiroshi Shimoda, Astri Nur Faizah, Yoshihiro Kaku, et al. 2024. “Identification and epidemiological study of an uncultured *flavivirus* from ticks using viral metagenomics and pseudoinfectious viral particles.” *Proceedings of the National Academy of Sciences of the United States of America* 121:e2319400121. https://doi.org/10.1073/pnas.2319400121
43. Jia, Na, Hong-Bo Liu, Xue-Bing Ni, Lesley Bell-Sakyi, Yuan-Chun Zheng, Ju-Liang Song, Jie Li, et al. 2019. “Emergence of human infection with Jingmen tick virus in China: A retrospective study.” *EBioMedicine* 43: 317-324. https://doi.org/10.1016/j.ebiom.2019.04.004
44. Simmonds, Peter, Mike J. Adams, Mária Benkő, Mya Breitbart, J. Rodney Brister, Eric B. Carstens, Andrew J. Davison, et al. 2017. “Consensus statement: Virus taxonomy in the age of metagenomics.” *Nature Reviews Microbiology* 15:161-168. https://doi.org/10.1038/nrmicro.2016.177
45. Kuhn, Jens H., Sergey V. Alkhovsky, Tatjana Avšič-Županc, Éric Bergeron, Felicity Burt, Koray Ergünay, Aura R Garrison, et al. 2024. “ICTV virus taxonomy profile: *Nairoviridae* 2024.” *Journal of General Virology* 105: 001974. https://doi.org/10.1099/jgv.0.001974
46. Sasaya, Takahide, Gustavo Palacios, Thomas Briese, Francesco Di Serio, Martin H. Groschup, Yutaro Neriya, Jinwon Song, and Yasuhiro Tomitaka. 2023. “ICTV virus taxonomy profile: *Phenuiviridae* 2023.” *Journal of General Virology* 104: 10.1099/jgv.0.001893. https://doi.org/10.1099/jgv.0.001893
47. Donaire, Livia, Jiatao Xie, Luca Nerva, Daohong Jiang, Shin-Yi Lee Marzano, Sead Sabanadzovic, Massimo Turina, and María A. Ayllón. 2024. “ICTV virus taxonomy profile: *Botourmiaviridae* 2024.” *Journal of General Virology* 105: 10.1099/jgv.0.002047. https://doi.org/10.1099/jgv.0.002047
48. Kuhn, Jens H., Nolwenn M. Dheilly, Sandra Junglen, Sofia Paraskevopoulou, Mang Shi, and Nicholas Di Paola. 2023. “ICTV virus taxonomy profile: *Jingchuvirales* 2023.” *Journal of General Virology* 104: 001924. https://doi.org/10.1099/jgv.0.001924
49. Valles, S. M., Y. Chen, A. E. Firth, D. M. A. Guérin, Y. Hashimoto, S. Herrero, J. R. de Miranda, E. Ryabov, and Ictv Report Consortium. 2017. “ICTV virus taxonomy profile: *Iflaviridae*.” *Journal of General Virology* 98: 527-528. https://doi.org/10.1099/jgv.0.000757
50. Harvey, Erin, and Edward C. Holmes. 2022. “Diversity and evolution of the animal virome.” *Nature Reviews Microbiology* 20: 321-334. https://doi.org/10.1038/s41579-021-00665-x
51. International Committee on Taxonomy of Viruses Executive Committee. 2020. “The new scope of virus taxonomy: partitioning the virosphere into 15 hierarchical ranks.” *Nature Microbiology* 5: 668-674. https://doi.org/10.1038/s41564-020-0709-x
52. Babayan, Simon A., Richard J. Orton, and Daniel G. Streicker. 2018. “Predicting reservoir hosts and arthropod vectors from evolutionary signatures in RNA virus genomes.” *Science* 362: 577-580. https://doi.org/10.1126/science.aap9072
53. Zhou, Hong, Lin Xu, and Weifeng Shi. 2023. “The human-infection potential of emerging tick-borne viruses is a global public health concern.” *Nature Reviews Microbiology* 21:215-217. https://doi.org/10.1038/s41579-022-00845-3
54. Zhang, Ming-Zhu, Cai Bian, Run-Ze Ye, Xiao-Ming Cui, Nan-Nan Yao, Ji-Hu Yang, Yan-Li Chu, et al. 2024. “A series of patients infected with the emerging tick-borne Yezo virus in China: an active surveillance and genomic analysis.” *The Lancet Infectious Diseases* S1473-3099(24)00616-9. https://doi.org/10.1016/S1473-3099(24)00616-9
55. Vechtova, Pavlina, Jarmila Sterbova, Jan Sterba, Marie Vancova, Ryan O. M. Rego, Martin Selinger, Martin Strnad, Maryna Golovchenko, Nataliia Rudenko, and Libor Grubhoffer. 2018. “A bite so sweet: the glycobiology interface of tick-host-pathogen interactions.” *Parasites & Vectors* 11: 594. https://doi.org/10.1186/s13071-018-3062-7
56. Sanchez-Vicente, Santiago, and Rafal Tokarz. 2023. “Tick-borne co-infections: challenges in molecular and serologic diagnoses.” *Pathogens* 12: 1371. https://doi.org/10.3390/pathogens12111371
57. Barillas-Mury, Carolina, Ribeiro José M. C., and Valenzuela Jesus G.. 2022. “Understanding pathogen survival and transmission by arthropod vectors to prevent human disease.” *Science* 377: eabc2757. https://doi.org/10.1126/science.abc2757
58. Cao, Duanfang, Bingting Ma, Ziyi Cao, Xinzheng Zhang, and Ye Xiang. 2023. “Structure of Semliki forest virus in complex with its receptor VLDLR.” *Cell* 186: 2208-2218.e15. https://doi.org/10.1016/j.cell.2023.03.032
59. Klaus, C., B. Hoffmann, U. Hering, B. Mielke, K. Sachse, M. Beer, and J. Süss. 2010. “Tick-borne encephalitis (TBE) virus prevalence and virus genome characterization in field-collected ticks (*Ixodes ricinus*) from risk, non-risk and former risk areas of TBE, and in ticks removed from humans in Germany.” *Clinical Microbiology and Infection* 16: 238-244. https://doi.org/10.1111/j.1469-0691.2009.02764.x
60. Wang, Shan-Shan, Jin-Yue Liu, Bao-Yu Wang, Wen-Jing Wang, Xiao-Ming Cui, Jia-Fu Jiang, Yi Sun, et al. 2023. “Geographical distribution of *Ixodes persulcatus* and associated pathogens: Analysis of integrated data from a China field survey and global published data.” *One Health* 16: 100508. https://doi.org/10.1016/j.onehlt.2023.100508
61. Zhao, Lin, Jie Li, Xiaoming Cui, Na Jia , Jiate Wei, Luoyuan Xia, Haitao Wang, et al. 2020. “Distribution of *Haemaphysalis longicornis* and associated pathogens: analysis of pooled data from a China field survey and global published data.” *The* *Lancet Planetary Health* 4: e320-9. https://doi.org/10.1016/S2542-5196(20)30145-5
62. Hou, Xin, Yong He, Pan Fang, Shi-Qiang Mei, Zan Xu, Wei-Chen Wu, Jun-Hua Tian, et al. 2024. “Using artificial intelligence to document the hidden RNA virosphere.” *Cell* 187: 6929-6942.e16. https://doi.org/10.1016/j.cell.2024.09.027
63. Mifsud, Jonathon C. O., Spyros Lytras, Michael R. Oliver, Kamilla Toon, Vincenzo A. Costa, Edward C. Holmes, and Joe Grove. 2024. “Mapping glycoprotein structure reveals *Flaviviridae* evolutionary history.” *Nature* 633:695-703. https://doi.org/10.1038/s41586-024-07899-8
64. Woolhouse, Mark. 2018. “Sources of human viruses.” *Science* 362: 524-525. https://doi.org/10.1126/science.aav4265
65. Carroll, Dennis, Peter Daszak, Nathan D. Wolfe, George F. Gao, Carlos M. Morel, Subhash Morzaria, Ariel Pablos-Méndez, Oyewale Tomori, and Jonna A. K. Mazet. 2018. “The global virome project.” *Science* 359:872-874. https://doi.org/10.1126/science.aap7463
